# Intimate partner violence among transgender people in Brazil: a cross-sectional study, 2015-2021

**DOI:** 10.1590/S2237-96222024v33e2024386.especial.en

**Published:** 2025-01-10

**Authors:** Diva Furtado Lacerda, Monarko Nunes de Azevedo

**Affiliations:** 1 Universidade Federal de Goiás, Instituto de Patologia Tropical e Saúde Pública, Programa de Pós-Graduação em Saúde Coletiva, Goiânia, GO, Brasil.; 2 Universidade Federal de Goiás, Instituto de Patologia Tropical e Saúde Pública, Departamento de Saúde Coletiva, Goiânia, GO, Brasil.

**Keywords:** Minorías de Género, Violencia por Parejas Íntimas, Vulnerabilidad Social, Interseccionalidad, Estudio Observacional, Gender Minorities, Intimate Partner Violence, Social Vulnerability, Intersectionality, Observational study

## Abstract

**Objective:**

To identify social vulnerability profiles of transgender people who have experienced intimate partner violence in Brazil and to assess the association with recurrent violence and referrals to support services.

**Methods:**

This was a cross-sectional study of reported cases of violence against transgender people in Brazil (2015-2021) utilizing data from SINAN/DATASUS. Sociodemographic profiles were defined using two-step cluster analysis and associations estimated by means of binary logistic regression, with odds ratios (OR) and confidence intervals (CI).

**Results:**

A total of 3,384 cases were reported, identifying five profiles. Recurrent violence was more frequent in the South region (71.1%), among individuals with elementary education (64.9%) and those with disabilities (74.0%) (p<0.001). Fewer referrals were observed in the North region (76.6%), among Asian and Indigenous people (74.5%) and among lesbians (81.6%) (p<0.05). No significant differences were found between the profiles (p>0.05).

**Conclusion:**

Social disparities influence intimate partner violence, but no significant differences were observed between the identified profiles.

## Study contributions

**Main results:**

Recurrent violence was observed in the South region, among individuals with elementary education and with physical disabilities or mental disorders. A lower frequency of referrals was found in the North region, among Indigenous people or people of Asian race /skin color, and lesbians.

**Implications for services:**

There is a need to strengthen strategies that promote equity in access to services, professional training and foster the development of culturally sensitive services, alongside raising awareness of the different forms of violence.

**Perspectives:**

Further research, considering elements not covered in this study, such as social support and systemic discrimination, may help elucidate additional factors related to patterns of recurrent violence.

## RESUMEN

**Objetivo:**

Identificar perfiles de vulnerabilidad social de personas transgénero víctimas de violencia por parte de parejas íntimas en Brasil; verificar la asociación con la repetición de violencia y derivación a servicios de atención.

**Métodos:**

Estudio transversal de casos denunciados de violencia contra personas trans en Brasil, utilizando datos del SINAN/DATASUS. Los perfiles sociodemográficos se delinearon mediante análisis de conglomerados en dos etapas; las asociaciones se estimaron mediante regresión logística binaria utilizando odds ratios (OR) e intervalos de confianza (IC).

**Resultados:**

Se reportaron 3.384 casos, delineándose cinco perfiles. La repetición de violencia fue más acentuada en el Sur (71,1%), entre personas con educación primaria (64,9%) y con discapacidad (74,0%) (p<0,001). Menos derivaciones en el Norte (76,6%), entre mujeres asiáticas e indígenas (74,5%), lesbianas (81,6%) (p<0,05).

**Conclusión:**

Disparidades sociales influyen en la violencia de pareja, pero no hay diferencias significativas entre los perfiles identificados.

## INTRODUCTION

Violence perpetrated against transgender people, particularly, is a complex and multifactorial phenomenon that encompasses events of various types and natures. ^1-6^ In addition to risk factors, it is essential to analyze it based on socio-environmental and political determinants and conditions, which are strongly associated with social inequalities. ^7,8^ Violence must be understood within the framework of social structures and power relations, which are reflected in cultural and behavioral issues. Furthermore, violence should be examined in the context of gender, class, and race/skin color relations. ^9,10^

Transgender people in Brazil experience violence on a daily basis. ^11,12^ Between October 2008 and September 2022, an alarming total of 1,741 transgender people and transvestites lost their lives due to violent acts. In the period from 2022 to 2023, Brazil recorded 96 transgender homicides, standing out as the country with the highest number of such violent cases. Europe, with 37 reported cases, and North America, with 26 cases, showed lower but still concerning figures.^11^ Between 2020 and 2021, physical violence against these people increased by 9.5%, while psychological violence rose by 20.4%. ^12^

The relevance of socioeconomic and demographic characteristics in shaping the context of violence experienced by LGBTQIAPN+ people is well recognized in the literature. ^1,2^ A racial perspective adds complexity when analyzing data on interpersonal violence against transgender people. From 2020 to 2021, Black transgender women faced a disproportionate rate of violence, accounting for 58% of cases, compared to 35% for White transgender women. Black transgender men experienced 56% of violent incidents, while White transgender men represented 40%. As for transvestites, the situation is even more alarming: 65% of violent incidents involved Black transvestites, compared to 31% for White transvestites. These data show how transphobic violence is closely linked to racial discrimination, intensifying threats faced by Black transgender people. ^12^

Regarding intimate partner violence directed at transgender people, a specific manifestation of violence occurs within relationships, where transphobia plays a significant role. This phenomenon encompasses various forms, including sexual, physical, verbal, psychological and financial violence. The severity of these aggressions is heightened among individuals with precarious socioeconomic conditions, substance abuse issues and those engaged in sex work, due to their greater exposure to historical processes that make them more vulnerable.^3,5^ Victims face particular challenges in seeking support, which makes it difficult to break the cycle of violence.^5^ It is worth highlighting that the discussion about intimate partner violence often focus on heterosexual and cisgender couples (people whose gender identity aligns with their biological sex), neglecting the experiences of sexual and gender minorities who face similar challenges.^3^

Analyzing the context of interpersonal violence against transgender people, based on the theoretical frameworks of intersectionality and vulnerability, offers a valuable perspective. These tools help healthcare professionals understand differences between individuals regarding gender, race/skin color, class, and other factors.^7^ This approach contributes to the analysis of public policies aimed at promoting equity, recognizing that needs may vary even within the transgender population. In clinical practice, intersectionality and vulnerability allow for the understanding that subjectivities differ based on prior experiences and social position. ^3-5^

The objectives of this study were to identify social vulnerability profiles of transgender people who have experienced intimate partner violence in Brazil and to assess the association with recurrent violence and referrals to victim support services.

## METHODS

### Study design

This was a cross-sectional study using secondary data.

### Setting

A quantitative approach was applied to the reported cases of intimate partner violence across all federative units in Brazil, taking into consideration records between 2015 and e 2021.

### Participants

The study population consisted of transvestites, transgender women and transgender men ,aged 20 to 59 years, based on records from the Notifiable Health Conditions Information System (*Sistema de Informações de Agravos de Notificação* - SINAN) ,available on the website of the Brazilian National Health System Information Technology Department (*Departamento de Informática do Sistema Único de Saúde* - DATASUS).

### Variables

The dependent variables of the study were recurrent violence and referrals to victim support services. The independent variables included age group, race/skin color, schooling, region of the country, physical disability or mental disorders, sexual orientation, and gender identity.

### Data sources and measurement

Data were obtained from SINAN, which has included fields for sexual orientation and gender identity in its notification form since the second half of 2014.^13^

Intimate partners were defined as individuals in an emotional intimate relationship with the victim, regardless of cohabitation, covering categories such as spouse, ex-spouse, boyfriend/girlfriend or ex-boyfriend/girlfriend. All forms of interpersonal violence described in the notification form were included, such as physical violence, psychological/moral violence, torture, sexual violence, financial/economic violence, etc.

The population was limited to transvestites, transgender women and transgender men because SINAN does not include other sexual orientations and identities.^13^

### Bias control

The completeness and consistency of the records, identifying missing data and temporal inconsistencies, were verified for data quality analysis. Critical variables, such as type of violence, sexual orientation and gender identity, were validated. Duplicate records were removed, and cases of self-inflicted violence were excluded.

### Study size

The study size was determined by considering the total number records available for the period, ensuring sample representativeness. This approach avoided random sampling, as the data were secondary and covered a significant universe of the target population.

### Statistical methods

Exploratory data analysis was performed using tests comparing the frequency of repeated episodes of intimate partners violence and cases referred to victim support services, which considered sociodemographic variables.

Social vulnerability profiles were identified through cluster analysis, which groups cases based on similarities, using sociodemographic variables to describe homogeneous clusters of transgender people. The following variables were used in the model: age group, race/skin color (grouping Black and mixed-race individuals as “Black”), schooling, region of the country, physical disability or mental disorders, sexual orientation and gender identity.

The two-step cluster method, available in SPSS ® 23.0 (Statistical Package for Social Science for Windows, Inc., USA), was used, as it is suitable for large databases with both continuous and categorical variables. This method performs agglomerative partitioning in two steps: first, pre-clusters are formed, and then they are regrouped to form final sub-profiles, with the optimal number of clusters determined by the Bayesian information criterion and distance measured by the log-likelihood, both standard options in the software.^14-16^

Sociodemographic profiles were described and compared in relation to the variables “recurrent violence” and “referral” through analysis of difference in proportions using Pearson’s chi- square with Bonferroni correction, at a significance level of 0.05.

Binary logistic regression analysis was used to verify the strength of the association between variables “recurrent violence” and “referral”, and the identified profiles at a significance level of 0.05. The odds ratio (OR) was calculated to quantify the strength of associations, along with 95% confidence intervals (CI) in order to assess the precision of the estimates. Profile A was comprised of Black, transgender women, aged 20 to 29 years, with high school education, from the Southeast region and without disabilities or disorders. This profile was used as the reference for the regression analysis because it corresponds to the profile with the lowest number of social vulnerability markers for violence.

### Ethical aspects

Ethical approval was not required as secondary data without participant identification were used.

## RESULTS

Between 2015 and 2021, a total of 3,384 cases of intimate partner violence against transgender people were reported in Brazil, distributed as follows: 717 in 2015; 910 in 2016; 2,800 in 2017; 157 in 2018; 147 in 2019; 140 in 2020; and 543 in 2021. The frequency of violence, analyzing sociodemographic characteristics separately, was higher among transgender women (82.0%), residents of the Southeast region (52.9%), aged 20 to 29 years (38.8%), Black people (54.7%), with elementary education (34.1%) and heterosexuals (70.7%). Reports of cases involving people with physical disabilities or mental disorders accounted for 4.6% of the population studied (Table 1).

**Table 1:** Sociodemographic characteristics of reported cases of intimate partner violence against transgender people in .Brazil, 2015-2021 ( N=3,384)

Sociodemographic characteristics	n(%)
**Region of the country**	
North	198(6.4)
Northeast	759(18.9)
Southeast	1,756(52.9)
South	415(14.1)
Midwest	256(7.7)
**Age group (years)**	
20-29	1,312(38.8)
30-39	1,235(36.5)
40-49	617(18.2)
50-59	220(6.5)
**Race/skin color**	
White	1,223(36.1)
Black	1,850(54.7)
Asian and Indigenous	48(1.4)
No information provided	263(7.8)
**Disability/disorder**	
Yes	155(4.6)
**Level of education**	
Up to elementary school	1,154(34.1)
Complete high school	1,033(30.5)
Complete higher education	220(6.5)
No information provided	977(28.9)
**Sexual orientation**	
Heterosexual	2,392(70.7)
Lesbian/ gay (homosexual)	631(18.6)
Bisexual	37(1,1)
No information provided	324(9.6)
**Gender identity**	
Transvestite	172(5.1)
Transgender woman	2.775(82.0)
Transgender man	437(12.9)

The exploratory analysis of Table 2 shows the results of the tests comparing the frequency of repeated episodes of intimate partner violence and the cases referred with the sociodemographic variables representing the social markers of difference. A higher frequency of recurrent cases was observed in the South region (p<0.001), individuals with elementary education (p<0.001) and with physical disabilities or mental disorders (p<0.001). There was no significant difference regarding age group (p=0.174), race/ skin color (p=0.048), sexual orientation (p=0.710) and gender identity (p=0.429). 

**Table 2:** Frequency of recurrent violence and referrals, according to the sociodemographic characteristics of reported cases of intimate partner violence against transgender people, Brazil, 2015-2021 ( N=3,384)

Variables	Recurrent violence		*p*-value	Referrals		*p*-value^a^
	Yes	No		Yes	No	
**Region of the country**			<0.001			<0.001
North	56.6	44.4		76.6	22.4	
Northeast	54.4	45.6		88.3	11.7	
Southeast	61.8	38.2		87.9	12.1	
South	71.1	28.9		84.7	10.6	
Midwest	57.0	43.0		87.3	15.3	
**Age group (years)**			0.174			0.869
20-29	58.4	41.6		87.3	12.7	
30-39	62.9	37.1		87.8	12.2	
40-49	60.6	39.4		86.4	13.6	
50-59	59.7	40.3		87.1	12.9	
**Race/skin color**			0.048			0.026
White	64.2	35.8		87.9	12.1	
Black	60.0	40.0		87.1	12.9	
Asian and Indigenous	68.2	31.8		74.5	25.5	
**Disability/disorder**			<0.001			0.704
Yes	74.0	26.1		86.4	13.6	
No	26.0	40.6		87.4	12.6	
**Level of education**			<0.001			0.257
Up to elementary school	64.9	35.1		87.8	12.2	
High school	61.2	38.8		86.2	13.8	
Higher education	60.3	39.7		84.7	15.3	
**Sexual orientation**			0.710			0.001
Heterosexual	59.9	40.1		88.2	11.8	
Lesbian	58.3	41.7		81.6	18.4	
Gay	64.1	35.9		90.6	9.4	
Bisexual	61.8	38.2		89.2	10.8	
**Gender identity**			0.429			0.418
Transvestite	55.6	44.4		84.5	15.5	
Transgender woman	60.8	39.2		87.6	12.4	
Transgender man	60.9	39.1		86.4	13.6	

a) Pearson’s chi -square with Bonferroni correction .

A greater frequency of reported cases of intimate partner violence that were referred, compared to those not referred, was identified, for all social markers of difference evaluated. It could be seen that transgender people from the North region (p<0.001), Asian or Indigenous people (p=0.026) and lesbians (p<0.001) received fewer referrals. There was no significant difference regarding age group (p=0.869), physical disability or mental disorders (p=0.704), schooling (p=0.257) and gender identity (p=0.418) (Table 2).

With regard to the two-step cluster method, the most important variables in predicting the profiles were “gender identity” and “sexual orientation”, followed by “race/skin color”, “level of education” and “region of the country”. “Age group” and “disability/disorder” were less influential in the formation of the profiles, which were outlined in five categories presented in Table 3.

**Table 3:** Outline of intimate partner violence reporting profiles among transgender people, according to sociodemographic characteristics. Brazil, 2015-2021 (n = 1,764)

Sociodemographic characteristics	Profile A 12.9% (228)	Profile B 27.2% (479)	Profile C 16.7% (295)	Profile D 13.4% (236)	Profile E 29.8% (526)
**Region of the country**					
North	0.0	3.1	2.0	5.0	12.0
Northeast	0.0	9.0	5.6	10.0	39.7
Southeast	100.0	45.9	82.7	64.0	35.0
South	0.0	35.0	5.0	12.0	5.3
Midwest	0.0	7.0	4.7	9.0	8.0
**Age group (years)**					
20-29	60.1	30.0	42.4	42.4	32.1
30-39	39.9	35.9	36.2	35.0	35.7
40-49	0.0	20.1	14.3	17.0	18.0
50-59	0.0	14.0	7.1	5.6	14.2
**Race/skin color**					
White	49.7	79.3	45.0	43.6	0.4
Black	51.3	16.0	52.9	56.4	99.6
Asian and Indigenous	0.0	4.7	2.1	0.0	0.0
**Level of education**					
Up to elementary school	0.0	49.9	48.8	48.3	68.6
High school	100.0	30.0	45.0	44.0	31.6
Higher education	0.0	20.1	6.2	7.7	0.0
**Disability/disorder**					
Without disability	100.0	87.1	95.2	92.8	100.0
With disability	0.0	12.9	4.8	7.2	0.0
**Sexual orientation**					
Heterosexual	100.0	99.2	0.0	71.2	100.0
Lesbian	0.0	0.8	91.2	0.0	0.0
Gay	0.0	0.0	0.0	28.8	0.0
Bisexual	0.0	0.0	8.8	0.0	0.0
**Gender identity**					
Transvestite	0.0	1.9	17.3	3.4	1.7
Transgender woman	100.0	98.1	82.7	0.0	97.3
Transgender man	0.0	0.0	0.0	93.6	1.0

The study identified five distinct profiles of transgender people: (A) women, heterosexual, predominantly Black, aged 20 to 29 years, from the Southeast region, with high school education and without disabilities; (B) women, heterosexual, of White race/skin color, from the Southeast region, aged 30 to 39 years, with elementary education and without disabilities; (C) women, lesbian, of Black race/skin color, from the Southeast region, with elementary education, aged 20 to 29 years and without disabilities; (D) men, gay , of Black race/skin color, from the Southeast region, with elementary education, aged 20 to 39 years and without disabilities; and (E) women, heterosexual, of Black race/skin color, from the Northeast region, with elementary education, aged 20 to 39 years and without disabilities ( Table 3) .

Profiles A, B, C, D and E predominantly presented a higher number of episodes of recurrent violence, ranging from 61.6% to 69.2%. All profiles showed a predominance of redirection to other victim support services. Notably, profile C showed the highest recurrent intimate partner violence, and profile A received referral or guidance in approximately 100.0% of cases (Table 4).

**Table 4:** Frequency (%) of recurrent violence and referrals, according to reporting profiles among transgender people. Brazil, 2015-2021 (n = 1,764)

Characteristics	Profile A^a^	Profile B^b^	Profile C^c^	Profile D^d^	Profile E^e^	*p*-value^f^
**Recurrent violence**						**0.375**
Occurred more than once	66.7	63.3	69.2	61.6	64.9	
Did not occurred more than once	33.3	36.7	30.8	38.4	35.1	
**Referral**						**0.277**
With referral	100.0	88.5	90.9	94.7	84.6	
Without referral	0.0	11.5	9.1	5.3	15.4	

a) **Profile A:** women, heterosexual, predominantly Black, aged 20 to 29 years, from the Southeast region, with high school education and without disabilities; b) **Profile B:** women, heterosexual, White, from the Southeast region, aged 30 to 39 years, with elementary education and without disabilities; c) **Profile C:** women, lesbians, Black, from the Southeast region, with elementary education, aged 20 to 29 years and without disabilities; d) **Profile D:** men, gay , Black, from the Southeast region, with elementary education, aged 20 to 39 years and without disabilities; e) **Profile E:** women, heterosexual, Black, from the Northeast region, with elementary education, aged 20 to 39 years and without disabilities; f) Pearson’s chi -square with Bonferroni correction .

No significant difference was found between the profiles regarding the frequency of recurrent violence (p=0.375) and referrals of reported cases (p=0.277) (Table 4). As for the likelihood recurrence of reported cases, the binary logistic regression analysis did not reveal statistically significant differences between the profiles when compared to profile A (Table 5).

**Table 5:** Odds ratios (OR) and 95% confidence intervals (95%CI) of recurrent violence according to reporting profiles among transgender people. Brazil, 2015-2021 (n = 1,764)

Variables	Profile B ^a^		Profile C ^b^		Profile D ^c^		Profile E ^d^	
**OR** **(95%CI)** ^e^	*p* **-value**	**OR (95%CI)** ^e^	*p* **-value**	**OR (95%CI)** ^e^	*p* **-value**	**OR (95%CI)** ^e^	*p* **-value**
**Recurrent violence**								
Occurred more than once	0.15 (0.312-4.292)	0.826	0.12 (0.165-4.777)	0.891	0.22 (0.343-4.519)	0.740	0.08 (0.273-4.293)	0.909
Did not occurred more than once	1.00		1.00		1.00		1.00	

a) **Profile B:** women, heterosexual, White, from the Southeast region, aged 30 to 39, with elementary education and without disabilities; b) **Profile C:** women, lesbians, Black, from the Southeast region, with elementary education, aged 20 to 29 and without disabilities; c) **Profile D:** men, gay , Black, from the Southeast region, with elementary education, aged 20 to 39 and without disabilities; d) **Profile E:** women, heterosexual, Black, from the Northeast region, with elementary education, aged 20 to 39 and without disabilities; e) For this analysis, profile A (women, heterosexual, predominantly Black, aged 20 TO 29, from the Southeast region, with high school education and without disabilities) was used as the reference category.

## DISCUSSION

The results of this study, based on the framework of intersectionality, align with the historical context of political and social rights, which were not guaranteed to transgender people at the time of their establishment.^7,17^ Different sexual orientation and gender identity exacerbate the issues of violence and unequal access to social rights for transgender people, even today, when taking into consideration the historical power relations in Brazil. These individuals face a dynamic of inequality, segregation and marginalization within the social structure, which plays a significant role of the production and reproduction of their identities.^18-20^

In this study, the absolute number of intimate partner violence cases against transgender people increased between 2015 and 2017, and sharply decreased in 2018, remained stable from 2018 to 2020, and increased again in 2021. The sharp drop in 2018 may have been influenced by several factors, such as changes in reporting policies, alterations in registration criteria, or variations in care practices. The implementation of more robust policies, such as the use of the Dial 100 (*Disque* 100), may have contributed to more systematic data collection on violence against the LGBTQIAPN+ population. It is worth highlighting that this reduction in the number of reported cases does not necessarily reflect an actual decrease in violence, and may be associated with underreporting, where many cases remain unreported due to fear of retaliations or lack of trust in authorities.

In 2020, COVID-19 control measures such as social distancing and lockdown may have affected both the reporting and incidence of violence. The pandemic led to increased stress, social isolation, and economic vulnerability, factors often associated with increased intimate partner violence. ^21-23^Although restrictions on movement may have hindered access to support services and case notifications, the increase observed in 2021 may be linked to the relaxation of COVID-19 control measures, which allowed for greater visibility and case notifications. ^21-23^

The Southeast region of Brazil, densely populated and urbanized, showed the highest number of intimate partner violence cases. Despite being the wealthiest region in the country, it faces high rates of notifications of intimate partner violence. This contrast reveals that economic wealth does not necessarily reduce violence. It also highlights that regional socioeconomic inequality can intensify the vulnerability of specific groups, increasing their exposure to violence. The highest frequency occurs among people aged 20 to 29, of Black race/skin color, those with elementary education, transgender women, and heterosexual women.^8,24^ The social markers of difference associated with a higher incidence reflect systemic discrimination patterns, while entrenched cultural norms contribute to an environment conducive to violence. ^7,19^

The high concentration of reported cases in certain regions may indicate greater visibility and efficiency in notification systems, which reflects increased awareness of the rights of transgender people and a greater willingness to report cases of violence. The Southeast region, for example, has a high number of reports, and may be more advanced in terms of recognizing and reporting cases of intimate partner violence. This situation highlights the need for specific and adapted strategies to address violence against transgender people, especially in regions with high notification rates.^25,26^

When the markers were evaluated separately, a statistical difference was observed in the frequency of repeated episodes of intimate partner violence against transgender people. In the South region, a higher frequency of repeated cases stood out, especially among those with elementary education and people with physical disabilities or mental disorders. In addition, a disparity was observed among groups of people from the North region, Asian or Indigenous individuals and lesbians who were less referred to support services.

The concentration of cases in these two Brazilian regions suggests geographic peculiarities, which indicates possible variations in prevention policies and support networks. ^8,24^ This shows limitations in the resources available in this area or geographic barriers that hinder access to support services. Furthermore, inequalities in access to support services and in the professionals’ perception of the severity of cases were observed. ^25-27^

The predominance of recurrent violence among people with elementary education underscores the specific challenges faced by individuals with lower education levels, such as reduced awareness of rights, difficulties in seeking help and prolonged exposure to environments conducive to violence.^19^

The lower frequency of referrals for Asian or Indigenous origin people may reflect cultural prejudice, a lack of understanding of the specific needs of these groups, or the absence of culturally sensitive services.^19,27-29^ The reduced frequency of referrals for lesbians suggests potential gaps in identifying and addressing specific needs of this community. This indicates the need for increased awareness of the different forms of violence affecting them or the presence of discrimination based on sexual orientation.^30^

These patterns emphasize the urgency of strategies that promote equity in access to support services. This includes training professionals, implementing culturally sensitive services, and raising awareness of the different forms of violence experienced by these specific groups. ^25-27^ Overcoming these barriers is essential to ensure that all individuals receive appropriate support when experiencing intimate partner violence.

In this study, five distinct profiles of transgender people were examined, each characterized by different sociodemographic aspects. It could be seen that all profiles showed more episodes of recurrent violence than non-recurrent violence, with profile C, which includes transgender women, from the Southeast region, of Black race/skin color, with elementary education and lesbians, standing out as the one with the highest number of recurrent violence episodes.

The analysis reveals variations in the relationship between individual characteristics and the experience of recurrent violence. The emphasis on profile C suggests that the intersectionality of oppressions plays a significant role .^7,18-20^ This indicates that specific combinations of identities, such as being a transgender woman, from the Southeast region, of Black race/skin color, with elementary education, and lesbian, may be associated with greater vulnerability to recurrent violence. Experiences of discrimination and violence may be intensified when multiple social markers of difference intersect. This highlights the complexity of social dynamics and the need for more sensitive and inclusive approaches in interventions and policies aimed at combating violence.^7,18-20^

It could be seen that all profiles were referred to other points within the victim support network. Profile A, consisting of a transgender woman, from the Southeast region, aged 20 to 29, of Black race skin color, with high school education, without physical disabilities or mental disorders and heterosexual, received the highest number referrals.

This finding indicates that profile A was more likely to be referred for support or guidance services. This may be attributed to a specific combination of factors, such as higher education, geographic characteristics, and the absence of physical disability or mental disorders, which may influence professionals’ perception of the need for referral.^18,19^ Understanding these specificities is crucial for developing more effective referral and support strategies, ensuring that assistance is appropriate and sensitive to the specific needs of each transgender person profile.

The confirmation that there was no significant difference between the profiles regarding the frequency of recurrent violence and referrals of reported cases is a relevant result. The binary logistic regression analysis found no statistically significant differences in the probability of recurrence of reported cases. This result underscores the complexity of the phenomenon and indicates that factors associated with recurrent violence do not vary significantly among the different profiles analyzed.

This lack of significant differences suggests that the individual and contextual characteristics considered in the profiles are not isolated determinants of recurrent violence. This indicates the need for a holistic approach to understanding and addressing intimate partner violence against transgender people. ^3,29^

The complexity of the phenomenon indicates that other factors, not addressed in the profiles, may have a crucial influence on recurrent violence. Elements such as social support, access to economic resources and systemic discrimination are mentioned as variables that play important roles. ^21,29^ Interpretating the results highlights the importance of considering a broader range of variables in understanding and preventing recurrent violence against transgender people. A holistic approach is essential for developing more effective strategies, which encompass both individual characteristics and various contextual factors that shape the dynamics of intimate partner violence.^3^

This study, although limited by aggregate data and biases in administrative records, is one of the first to address intimate partner violence against transgender people during the COVID-19 pandemic. ^21^ The political and social issues faced by LGBTQIAPN+ people remain challenging, with an increase and fluctuation in intimate partner violence cases over the years, influenced by notification policies and the pandemic. Regional disparities highlight the need for specific approaches, and the lack of significant differences between profiles in terms of recurrent violence and referrals points to the importance of a holistic approach that considers social support and systemic discrimination. The study contributes to the initial understanding of the phenomenon and reinforces the need for ongoing research and interventions that are sensitive to its complexities.

## References

[1] Peixoto VB (2018). Violência contra LGBTs no Brasil: premissas históricas da violação no Brasil. PERI.

[2] Prado AJ, de Sousa MF (2017). Políticas Públicas e a Saúde da População LGBT: Uma revisão integrativa. Tempus Actas de Saúde Coletiva.

[3] da Silva ICB, de Araújo EC, Santana AD da S, Moura JW da S, Ramalho MN de A, de Abreu PD (2022). A violência de gênero perpetrada contra mulheres trans. Rev.Bras Enferm.

[4] Miskolci R, Signorelli MC, Canavese D, Teixeira F do B, Polidoro M, Moretti-Pires RO, de Souza MHT, Pereira PPG (2022). Desafios da saúde da população LGBTI+ no Brasil: uma análise do cenário por triangulação de métodos. Cien Saude Colet.

[5] Sena AGN, Souto KMB (2017). Avanços e desafios na implementação da Política Nacional de Saúde Integral LGBT. TEMPUS.

[6] Brasil (2001). Política Nacional de Redução da Morbimortalidade por Acidentes e Violências.

[7] Luiz O do C, Couto MT, Oliveira E de, Separavich MA (2020). Inequality in health, social determinants, and intersectionality: a systematic review/Desigualdade em saúde, determinantes sociais, e interseccionalidade: uma revisão sistemática. Braz. J. Hea. Rev.

[8] Marzulo EP, Heck MA, Filippi EE (2020). Desigualdades socioeconômicas no Brasil. DRd - Desenvolvimento Regional em debate.

[9] Minayo MC de S (2006). Violência e Saúde.

[10] Krug E, Dahlberg L, Mercy J, Zwi A, Lozano R (2002). The world report on violence and health.

[11] (2022). Transrespect versus Transphobia Worldwide, Trans Murder Monitoring - TMM.

[12] Cerqueira D, Bueno S (2023). Atlas da Violência 2023.

[13] Brasil (2016). VIVA, Instrutivo de Notificação de Violência Interpessoal e Autoprovocada.

[14] Figueiredo DB, Silva JA da, Rocha EC da (2012). Classificando regimes políticos utilizando análise de conglomerados. Opin Publica.

[15] Bacher J, Wenzig K, Vogler M (2004). SPSS TwoStep Cluster-a first evaluation.

[16] Chan Y (2005). Biostatistics 304. Cluster Analysis. Singapore Med J.

[17] Couto MT, de Oliveira E, Separavich MAA, Luiz O do C (2019). The feminist perspective of intersectionality in the field of public health: A narrative review of the theoretical-methodological literature. Salud Colect.

[18] Nogueira FJ de S, Leitão ES de F, Silva ECS da (2021). Interseccionalidades na Experiência de Pessoas Trans nos Serviços de Saúde. PSSA.

[19] Goulart VP, Nardi HC (2022). Vidas inimigas, necropolítica e interseccionalidade: da exclusão na educação ao suicídio/assassinato de pessoas trans. Revista Entreideias: Educação, Cultura e Sociedade.

[20] Shelton SA, Lester AOS (2020). A narrative exploration of the importance of intersectionality in a Black trans woman’s mental health experiences. Int J Transgend Health.

[21] Evans ML, Lindauer M, Farrell ME (2020). A Pandemic within a Pandemic, Intimate Partner Violence during Covid-19. New England Journal of Medicine.

[22] Marques ALM, Sorentino I da S, Rodrigues JL, Machin R, Oliveira E de, Couto MT (2021). O impacto da Covid-19 em grupos marginalizados: contribuições da interseccionalidade como perspectiva teórico-política. Interface - Comunicação, Saúde, Educação.

[23] Braga LHR, Menezes CS, Martins IV, Da Silva JDP, Torres JL (2022). Fatores associados à piora no estilo de vida durante a pandemia de Covid-19 na população brasileira de lésbicas, gays, bissexuais, transexuais, travestis e identidades relacionadas: estudo transversal. Epidemiologia e Serviços de Saúde.

[24] De Albuquerque MV, Viana AL d’Ávila LD, De Lima LD, Ferreira MP, Fusaro ER, Iozzi FL (2017). Regional health inequalities: changes observed in Brazil from 2000-2016. Cien Saude Colet.

[25] Minayo MCDS, De Souza ER, Da Silva MMA, De Assis SG (2018). Institucionalização do tema da violência no SUS: avanços e desafios. Cien Saude Colet.

[26] De Oliveira APC, Gabriel M, Dal Poz MR, Dussault G (2017). Challenges for ensuring availability and accessibility to health care services under Brazil’s Unified Health System (SUS). Cien Saude Colet.

[27] Jesus MKMR de, Moré IAA, Querino RA, Oliveira VH de (2023). Experiências de mulheres transexuais no sistema de saúde: visibilidade em direção à equidade. Interface - Comunicação, Saúde, Educação.

[28] Vitral Pinto II, Suely de Araújo Andrade SI, Lofego Rodrigues III L, Aline Siqueira Santos MI, Melo Arruda Marinho MI, Andrade Benício LI (2020). Profile of notification of violence against Lesbian, Gay, Bisexual, Transvestite and Transsexual people recorded in the National Information System on Notifiable Diseases, Brazil, 2015-2017. Rev Bras Epidemiol.

[29] Félix e Silva AL, Mendonça CCC, Viana BA, Souza IWC da S, Azevedo MN (2023). Violência interpessoal contra adolescentes LGBT: Uma perspectiva ampliada sobre tendências, contextos regionais e desafios emergentes. Revista de Antropologia da UFSCar.

[30] Santana ADS, Lima MS de, Moura JWS, Vanderley ICS, Araújo EC de (2020). Dificuldades no acesso aos serviços de saúde por lésbicas, gays, bissexuais e transgêneros. Rev Enferm UFPE online.

