# Supplementary material for: Intimate partner violence among transgender people in Brazil: a cross-sectional study, 2015-2021
Source: Epidemiol Serv Saude. 2025 Jan 10;33(spe1):e2024386. doi: 10.1590/S2237-96222024v33e2024386.especial.en (PMC11734655; doi:10.1590/S2237-96222024v33e2024386.especial.en)
Supplement: Translation

# Translation: Violência por parceiros íntimos em pessoas transgênero no Brasil: estudo transversal, 2015-2021

**DOI:** 10.1590/S2237-96222024v33e2024386.especial.pt

**Authors:** Diva Furtado Lacerda, Monarko Nunes de Azevedo

**Affiliations:** 1 Universidade Federal de Goiás, Instituto de Patologia Tropical e Saúde Pública, Programa de Pós-Graduação em Saúde Coletiva, Goiânia, GO, Brasil.; 2 Universidade Federal de Goiás, Instituto de Patologia Tropical e Saúde Pública, Departamento de Saúde Coletiva, Goiânia, GO, Brasil.

**Keywords:** Minorias de Gênero, Violência por Parceiros íntimos, Vulnerabilidade Social, Interseccionalidade, Estudo Observacional

## Abstract

**Objetivo:**

Identificar perfis de vulnerabilidade social de pessoas transgênero vítimas de violência por parceiros íntimos no Brasil e verificar a associação com a repetição da violência e encaminhamentos para serviços de atendimento.

**Métodos:**

Estudo transversal dos casos notificados de violência contra pessoas trans no Brasil (2015-2021) usando dados do SINAN/DATASUS. Perfis sociodemográficos foram delineados por análise de cluster em duas etapas e associações estimadas por regressão logística binária utilizando a razão de chances (*odds ratio*, OR) e intervalos de confiança (IC).

**Resultados:**

Foram notificados 3.384 casos, delineando cinco perfis. A repetição da violência foi mais frequente no Sul (71,1%), entre pessoas com ensino fundamental (64,9%) e com deficiência (74,0%) (p<0,001). Menos encaminhamentos no Norte (76,6%), entre amarelas e indígenas (74,5%) e lésbicas (81,6%) (p<0,05). Não houve diferenças significativas entre os perfis (p>0,05).

**Conclusão:**

Disparidades sociais influenciam a violência por parceiros íntimos, mas não há diferenças significativas entre os perfis identificados.

## Contribuições do estudo

**Principais resultados:**

Violência de repetição foi observada na região Sul, pessoas com ensino fundamental e com deficiência física ou transtorno mental. Menor frequência de encaminhamentos na região Norte, pessoas indígenas ou de cor/raça amarela, e pessoas lésbicas.

**Implicações para os serviços:**

É necessário fortalecer estratégias que promovam equidade no acesso aos serviços, capacitação profissional e promoção de serviços culturalmente sensíveis e a conscientização sobre as diferentes formas de violência.

**Perspectivas:**

Pesquisas de maior alcance, considerando elementos não contemplados como apoio social e discriminação sistêmica potencialmente elucidarão aspectos adicionais sobre os perfis de repetição.

## INTRODUÇÃO

A violência perpetrada contra as pessoas transgênero, especificamente, é um fenômeno de conceituação complexa e multicausal que abrange eventos de tipologias e naturezas diversas.^1-6^ Além dos fatores de risco, é necessário analisá-la a partir dos determinantes e condicionantes socioambientais e políticos, fortemente associados às desigualdades sociais.^7,8^ É crucial entender a violência nas estruturas sociais e nas relações de poder, que se refletem em questões culturais e comportamentais. A violência também deve ser examinada à luz das relações de gênero, classe e raça/cor da pele.^9,10^

As pessoas transgênero vivenciam cotidianamente situações de violência no Brasil.^11,12^ Entre outubro de 2008 e setembro de 2022, o alarmante total de 1.741 pessoas transexuais e travestis perderam suas vidas devido a atos violentos no país. No período 2022-2023, o Brasil registrou 96 assassinatos de pessoas trans, destacando-se como o país com o maior número de casos de violência nesse quesito. A Europa, com 37 casos registrados, e a América do Norte, com 26 casos, apresentaram números menores, mas ainda preocupantes.^11^ No intervalo entre 2020 e 2021, a violência física contra essas pessoas aumentou 9,5%, enquanto a violência psicológica cresceu 20,4%.^12^

A relevância das características socioeconômicas e demográficas na determinação do contexto da violência experimentada por pessoas LGBTQIAPN+ é devidamente reconhecida na literatura.^1,2^ A perspectiva racial revela uma complexidade a mais ao analisar os dados sobre a violência interpessoal contra pessoas trans. Entre 2020 e 2021, as mulheres transexuais negras enfrentaram uma taxa desproporcional de violência, correspondendo a 58% dos casos, em comparação com 35% para as mulheres transexuais brancas. Os homens trans negros sofreram 56% dos incidentes de violência, enquanto os homens trans brancos representaram 40%. No caso das travestis, a situação é ainda mais alarmante: 65% dos incidentes de violência envolvem travestis negras, em contraste com 31% das travestis brancas. Esses dados evidenciam como a violência transfóbica está bastante ligada à discriminação racial, intensificando as ameaças às pessoas trans negras.^12^

Na violência praticada por parceiros íntimos, direcionada a pessoas transgênero, há uma expressão específica de violência, que impacta esses indivíduos em relacionamentos, nos quais a transfobia desempenha um papel significativo. Esse fenômeno engloba diversas manifestações, que incluem violência sexual, física, verbal, psicológica e financeira. A intensidade dessas agressões é agravada entre aqueles em condições socioeconômicas precárias, usuários abusivos de substâncias psicoativas e profissionais do sexo, devido à maior exposição a processos históricos que os tornam mais vulneráveis.^3,5^ As vítimas enfrentam desafios particulares ao buscar apoio, o que torna a interrupção do ciclo de violência uma tarefa complexa.^5^ É importante observar que a discussão sobre violência praticada por parceiros íntimos é frequentemente centrada nos casais heterossexuais e cisgênero (pessoas cuja identidade de gênero coincide com o sexo biológico), negligenciando as experiências das minorias sexuais e de gêneros que enfrentam desafios semelhantes.^3^

A análise do panorama da violência interpessoal contra pessoas transgênero, a partir dos referenciais teóricos da interseccionalidade e da vulnerabilidade, oferece uma perspectiva valiosa. Essas ferramentas auxiliam os profissionais de saúde a compreender as diferenças existentes entre indivíduos em relação a gênero, raça/cor da pele, classe, entre outros marcadores.^7^ Esse enfoque contribui para a análise de políticas públicas que promovam equidade, reconhecendo-se que as necessidades podem variar entre pessoas do mesmo segmento trans. Na prática clínica, a interseccionalidade e a vulnerabilidade permitem entender que as subjetividades diferem com base nas experiências prévias e na posição social.^3-5^

Os objetivos deste trabalho foram o de identificar perfis de vulnerabilidade social de pessoas transgênero vítimas de violência praticada por parceiros íntimos no Brasil e verificar a associação com a repetição da violência e os encaminhamentos para os serviços de atendimento às vítimas.

## MÉTODOS

### Delineamento 

Este trata-se de um estudo transversal com dados secundários.

### Contexto

Foi realizada uma abordagem quantitativ**a** dos casos notificados de violência praticada por parceiros íntimos de todas as unidades federativas do Brasil, considerando registros entre 2015 e 2021.

### Participantes

A população do estudo foi constituída por travestis, mulheres transexuais e homens transexuais, com idade entre 20 e 59 anos, a partir dos registros do Sistema de Informações de Agravos de Notificação (SINAN), disponíveis no sítio eletrônico do Departamento de Informática do Sistema Único de Saúde (DATASUS).

### Variáveis

As variáveis dependentes do estudo foram violência de repetição e encaminhamento para os serviços de atendimento às vítimas. As variáveis independentes incluíram faixa etária, raça/cor da pele, nível de escolaridade, região do país, deficiência física ou transtorno mental, orientação sexual e identidade de gênero. 

### Fontes de dados e mensuração

Os dados foram obtidos do SINAN, que inclui campos de orientação sexual e identidade de gênero na sua ficha de notificação desde o segundo semestre de 2014.^13^

Consideraram-se parceiros íntimos os indivíduos que mantêm uma relação íntima de afeto com a vítima, independentemente de coabitação, abarcando-se as categorias de cônjuge, ex-cônjuge, namorado(a) ou ex-namorado(a). Abrangeram-se todos os tipos de violência interpessoal descritos na ficha de notificação, incluindo violência física, violência psicológica/moral, tortura, violência sexual, violência financeira/econômica etc.

A definição da população apenas de travestis, mulheres transexuais e homens transexuais se deu pelo fato de o SINAN não contemplar as demais orientações sexuais e identidades existentes.^13^

### Controle de viés

A completude e a consistência dos registros, identificando dados faltantes e incoerências temporais, foram verificadas para a análise da qualidade dos dados. Validaram-se variáveis críticas, como tipo de violência, orientação sexual e identidade de gênero. Registros duplicados foram eliminados, e casos de violência autoprovocada, excluídos. 

### Tamanho do estudo

O tamanho do estudo foi definido considerando a totalidade de registros disponíveis para o período, garantindo a representatividade da amostra. Essa abordagem evitou a amostragem aleatória, uma vez que os dados eram secundários e abrangiam um universo significativo da população alvo.

### Métodos estatísticos

A análise exploratória dos dados foi realizada por meio de testes de comparação da frequência dos episódios de repetição da violência praticada por parceiros íntimos e dos casos encaminhados para serviços de atendimento às vítimas, que consideraram as variáveis sociodemográficas. 

Os perfis de vulnerabilidade social foram identificados através de uma análise de *cluster*, que agrupa casos com base em semelhanças, utilizando-se variáveis sociodemográficas para descrever agrupamentos homogêneos de pessoas transgênero. No modelo, foram usadas as variáveis: faixa etária, raça/cor da pele (agrupando pretos e pardos sob a denominação “negros”), escolaridade, região do país, deficiência física ou transtorno mental, orientação sexual e identidade de gênero. 

O método de Cluster em duas etapas, disponível no SPSS® 23.0 (Statistical Package for Social Science for Windows, Inc., USA), foi empregado, pois é adequado para bases de dados grandes e variáveis contínuas e categóricas. Esse método realiza particionamentos aglomerativos em duas etapas: primeiro, formam-se pré-*clusters* e, em seguida, reagrupam-se para formar subperfis finais, com o número ideal de agrupamentos determinado pelo critério de informação bayesiano e a distância medida pelo log-verossimilhança, ambas opções padrão do programa.^14-16^

Os perfis sociodemográficos foram descritos e comparados em relação às variáveis “violência de repetição” e “encaminhamento” por meio da análise de diferença de proporções usando-se o qui-quadrado de Pearson com a correção de Bonferroni, ao nível de significância de 0,05. 

A análise de regressão logística binária foi utilizada para verificar a força da associação entre as variáveis “violência de repetição” e “encaminhamento”, e os perfis delineados ao nível de significância de 0,05. A razão de chances (*odds ratio*, OR) foi calculada para quantificar a força das associações, juntamente com os intervalos de confiança (IC) de 95% para avaliar a precisão das estimativas. O perfil A foi composto por transexuais mulheres negras, heterossexuais, com idade entre 20 e 29 anos, ensino médio, da região Sudeste e sem deficiência ou transtorno. Esse perfil foi utilizado como referência para a análise de regressão por corresponder ao perfil com menor número de marcadores sociais de vulnerabilidade para a violência.

### Aspectos éticos

Não houve necessidade de apreciação ética por empregar dados secundários e sem identificação dos participantes. 

## RESULTADOS

Entre 2015 e 2021, foram notificados 3.384 casos de violência praticada por parceiros íntimos contra pessoas transgênero no Brasil, distribuídos da seguinte forma: 717 em 2015; 910 em 2016; 2.800 em 2017; 157 em 2018; 147 em 2019; 140 em 2020; e 543 em 2021. A frequência da violência, analisando separadamente as características sociodemográficas, foi mais elevada em mulheres transexuais (82,0%), residentes na região Sudeste (52,9%), na faixa etária de 20 a 29 anos (38,8%), pessoas negras (54,7%), com ensino fundamental (34,1%) e heterossexuais (70,7%). As notificações de casos de pessoas com deficiência física ou transtorno mental representaram 4,6% da população estudada (Tabela 1).

**Tabela 1:** Características sociodemográficas dos casos notificados de violência por parceiros íntimos em pessoas transgênero. Brasil, 2015-2021 (N=3.384)

Caracteristicas sociodemográficas	n(%)
**Região do país**	
Norte	198(6,4)
Nordeste	759(18,9)
Sudeste	1.756(52,9)
Sul	415(14,1)
Centro-Oeste	256(7,7)
**Faixa etária (anos)**	
20-29	1.312(38,8)
30-39	1.235(36,5)
40-49	617(18,2)
50-59	220(6,5)
**Raça/cor da pele**	
Brancos	1.223(36,1)
Negros	1.850(54,7)
Amarelos e indígenas	48(1,4)
Sem informação	263(7,8)
**Deficiência/transtorno**	
Sim	155(4,6)
**Nível de escolaridade**	
Até ensino fundamental	1.154(34,1)
Ensino médio completo	1.033(30,5)
Ensino superior completo	220(6,5)
Sem informação	977(28,9)
**Orientação sexual**	
Heterossexual	2.392(70,7)
Lésbica/*gay* (homossexual)	631(18,6)
Bissexual	37(1,1)
Sem informação	324(9,6)
**Identidade de gênero**	
Travesti	172(5,1)
Transexual mulher	2.775(82,0)
Transexual homem	437(12,9)

A análise exploratória da Tabela 2 apresenta os resultados dos testes de comparação da frequência dos episódios de repetição da violência praticada por parceiros íntimos e dos casos encaminhados com as variáveis sociodemográficas representativas dos marcadores sociais da diferença. Notou-se maior frequência de repetição dos casos na região Sul (p<0,001), pessoas com ensino fundamental (p<0,001) e com deficiência física ou transtorno mental (p<0,001). Não houve diferença significativa em relação à faixa etária (p=0,174), raça/cor da pele (p=0,048), orientação sexual (p=0,710) e identidade de gênero (p=0,429). 

**Tabela 2:** Frequência da violência de repetição e encaminhamento, segundo as características sociodemográficas dos casos notificados de violência por parceiros íntimos em pessoas transgênero. Brasil, 2015-2021 (N=3.384)

Variáveis	Repetição da violência		*p*-valor^a^	Encaminhamento		*p*-valor^a^
	Sim	Não		Sim	Não	
**Região do país**			<0,001			<0,001
Norte	56,6	44,4		76,6	22,4	
Nordeste	54,4	45,6		88,3	11,7	
Sudeste	61,8	38,2		87,9	12,1	
Sul	71,1	28,9		84,7	10,6	
Centro-Oeste	57,0	43,0		87,3	15,3	
**Faixa etária (anos)**			0,174			0,869
20-29	58,4	41,6		87,3	12,7	
30-39	62,9	37,1		87,8	12,2	
40-49	60,6	39,4		86,4	13,6	
50-59	59,7	40,3		87,1	12,9	
**Raça/cor da pele**			0,048			0,026
Brancos	64,2	35,8		87,9	12,1	
Negros	60,0	40,0		87,1	12,9	
Amarelos e indígenas	68,2	31,8		74,5	25,5	
**Deficiência/transtorno**			<0,001			0,704
Sim	74,0	26,1		86,4	13,6	
Não	26,0	40,6		87,4	12,6	
**Nível de escolaridade**			<0,001			0,257
Até ensino fundamental	64,9	35,1		87,8	12,2	
Ensino médio	61,2	38,8		86,2	13,8	
Ensino superior	60,3	39,7		84,7	15,3	
**Orientação sexual**			0,710			0,001
Heterossexual	59,9	40,1		88,2	11,8	
Lésbica	58,3	41,7		81,6	18,4	
Gay	64,1	35,9		90,6	9,4	
Bissexual	61,8	38,2		89,2	10,8	
**Identidade de gênero**			0,429			0,418
Travesti	55,6	44,4		84,5	15,5	
Transexual mulher	60,8	39,2		87,6	12,4	
Transexual homem	60,9	39,1		86,4	13,6	

a) Qui-quadrado de Pearson com a correção de Bonferroni.

Identificou-se maior frequência de casos notificados de violência praticada por parceiros íntimos encaminhados, em comparação aos casos sem encaminhamento, para todos os marcadores sociais da diferença avaliados. Observou-se que pessoas transgênero da região Norte (p<0,001), amarelas ou indígenas (p=0,026) e lésbicas (p<0,001) receberam menos encaminhamentos. Não houve diferença significativa em relação à faixa etária (p=0,869), deficiência física ou transtorno mental (p=0,704), nível de escolaridade (p=0,257) e identidade de gênero (p=0,418) (Tabela 2).

No método de Cluster em duas etapas, as variáveis de maior importância na predição dos perfis foram “identidade de gênero” e “orientação sexual”, seguidas por “raça/cor da pele”, “nível de escolaridade” e “região do país”. “Faixa etária” e “deficiência/transtorno” tiveram menor peso na formação dos perfis, que foram delineados em cinco categorias apresentadas na Tabela 3.

**Tabela 3:** Delineamento dos perfis de notificação de violência por parceiros íntimos em pessoas transgênero, segundo as características sociodemográficas. Brasil, 2015-2021 (n = 1.764)

Características sociodemográficas	Perfil A 12,9% (228)	Perfil B 27,2% (479)	Perfil C 16,7% (295)	Perfil D 13,4% (236)	Perfil E 29,8% (526)
**Região do país**					
Norte	0,0	3,1	2,0	5,0	12,0
Nordeste	0,0	9,0	5,6	10,0	39,7
Sudeste	100,0	45,9	82,7	64,0	35,0
Sul	0,0	35,0	5,0	12,0	5,3
Centro-Oeste	0,0	7,0	4,7	9,0	8,0
**Faixa etária (anos)**					
20-29	60,1	30,0	42,4	42,4	32,1
30-39	39,9	35,9	36,2	35,0	35,7
40-49	0,0	20,1	14,3	17,0	18,0
50-59	0.0	14,0	7,1	5,6	14,2
**Raça/cor da pele**					
Brancos	49,7	79,3	45,0	43,6	0,4
Negros	51,3	16,0	52,9	56,4	99,6
Amarelos e indígenas	0,0	4,7	2,1	0,0	0,0
**Nível de escolaridade**					
Até ensino fundamental	0,0	49,9	48,8	48,3	68,6
Ensino médio	100,0	30,0	45,0	44,0	31,6
Ensino superior	0,0	20,1	6,2	7,7	0,0
**Deficiência/transtorno**					
Sem deficiência	100,0	87,1	95,2	92,8	100,0
Com deficiência	0,0	12,9	4,8	7,2	0,0
**Orientação sexual**					
Heterossexual	100,0	99,2	0,0	71,2	100,0
Lésbica	0,0	0,8	91,2	0,0	0,0
Gay	0,0	0,0	0,0	28,8	0,0
Bissexual	0,0	0,0	8,8	0,0	0,0
**Identidade de gênero**					
Travesti	0,0	1,9	17,3	3,4	1,7
Transexual mulher	100,0	98,1	82,7	0,0	97,3
Transexual homem	0,0	0,0	0,0	93,6	1,0

O estudo identificou cinco perfis distintos de transexuais: (A) mulheres, heterossexuais, majoritariamente negras, idade entre 20 e 29 anos, do Sudeste, com ensino médio e sem deficiência; (B) mulheres, heterossexuais, brancas, do Sudeste, com idade entre 30 e 39 anos, ensino fundamental e sem deficiência; (C) mulheres, lésbicas, negras, do Sudeste, com ensino fundamental, idade entre 20 e 29 anos e sem deficiência; (D) homens, *gays*, negros, do Sudeste, com ensino fundamental, idade entre 20 e 39 anos e sem deficiência; e (E) mulheres, heterossexuais, negras, do Nordeste, com ensino fundamental, idade entre 20 e 39 anos e sem deficiência (Tabela 3).

Os perfis A, B, C, D e E apresentaram, em sua maioria, maiores episódios de repetição da violência, variando de 61,6% a 69,2%. Todos os perfis apresentaram predominância de redirecionamento para outros pontos da rede de assistência à vítima de violência. Destacam-se o perfil C, que apresentou mais repetição da violência praticada por parceiros íntimos, e o perfil A, que recebeu encaminhamento ou orientação em aproximadamente 100,0% dos casos (Tabela 4).

**Tabela 4:** Frequência (%) da violência de repetição e encaminhamentos, segundo os perfis de notificação em pessoas transgênero. Brasil, 2015-2021 (n = 1.764)

Características	Perfil A^a^	Perfil B^b^	Perfil C^c^	Perfil D^d^	Perfil E^e^	*p*-valor^f^
**Violência de repetição**						0,375
Ocorreu outras vezes	66,7	63,3	69,2	61,6	64,9	
Não ocorreu outras vezes	33,3	36,7	30,8	38,4	35,1	
**Encaminhamento**						0,277
Com encaminhamento	100,0	88,5	90,9	94,7	84,6	
Sem encaminhamento	0,0	11,5	9,1	5,3	15,4	

a) **Perfil A:** mulheres, heterossexuais, majoritariamente negras, idade entre 20 e 29 anos, do Sudeste, com ensino médio e sem deficiência; b) **Perfil B:** mulheres, heterossexuais, brancas, do Sudeste, com idade entre 30 e 39 anos, ensino fundamental e sem deficiência; c) **Perfil C:** mulheres, lésbicas, negras, do Sudeste, com ensino fundamental, idade entre 20 e 29 anos e sem deficiência; d) **Perfil D:** homens, *gays*, negros, do Sudeste, com ensino fundamental, idade entre 20 e 39 anos e sem deficiência; e) **Perfil E:** mulheres, heterossexuais, negras, do Nordeste, com ensino fundamental, idade entre 20 e 39 anos e sem deficiência; f) Qui-quadrado de Pearson com a correção de Bonferroni.

Não houve diferença significativa entre os perfis delineados em relação à frequência da repetição da violência (p=0,375) e dos encaminhamentos dos casos notificados (p=0,277) (Tabela 4). Quanto à chance de os casos notificados ocorrerem novamente, a análise de regressão logística binária não revelou diferenças estatisticamente significativas entre os perfis em comparação ao perfil A (Tabela 5).

**Tabela 5:** Razões de chance (*odds ratio*, OR) e intervalos de confiança de 95% (IC95%) da violência de repetição pelos perfis notificação em pessoas transgênero. Brasil, 2015-2021 (n = 1.764)

Variáveis	Perfil B^a^		Perfil C^b^		Perfil D^c^		Perfil E^d^	
	OR (IC95%) ^e^	*p* -valor	OR (IC95%) ^e^	*p* -valor	OR (IC95%) ^e^	*p* -valor	OR (IC95%) ^e^	*p* -valor
**Violência de repetição**								
Ocorreu outras vezes	0,15 (0,312-4,292)	0,826	0,12 (0,165-4,777)	0,891	0,22 (0,343-4,519)	0,740	0,08 (0,273-4,293)	0,909
Não ocorreu outras vezes	1,00		1,00		1,00		1,00	

a) **Perfil B:** mulheres, heterossexuais, brancas, do Sudeste, com idade entre 30 e 39 anos, ensino fundamental e sem deficiência; b) **Perfil C:** mulheres, lésbicas, negras, do Sudeste, com ensino fundamental, idade entre 20 e 29 anos e sem deficiência; c) **Perfil D:** homens, *gays*, negros, do Sudeste, com ensino fundamental, idade entre 20 e 39 anos e sem deficiência; d) **Perfil E:** mulheres, heterossexuais, negras, do Nordeste, com ensino fundamental, idade entre 20 e 39 anos e sem deficiência; e) Para essa análise, a categoria de referência foi o perfil A (mulheres, heterossexuais, majoritariamente negras, idade entre 20 e 29 anos, do Sudeste, com ensino médio e sem deficiência).

## DISCUSSÃO

Os resultados deste estudo, segundo o referencial da interseccionalidade, vão ao encontro da história dos direitos, nos âmbitos político e social, os quais não foram garantidos às pessoas transgênero, no momento da instauração dos direitos.^7,17^ As distintas orientações sexuais e identidades de gênero aprofundam o problema da violência e diferença ao acesso de pessoas trans aos direitos sociais, ainda na contemporaneidade, quando se toma a historicidade das relações de poder no Brasil. Esses sujeitos encontram, na estrutura social, uma dinâmica de situações de desigualdade, segregação e marginalização, que constituem parte importante da produção e reprodução de suas identidades.^18-20^

Neste estudo, o número absoluto da violência praticada por parceiros íntimos contra pessoas trans aumentou de 2015 a 2017, diminuiu bruscamente em 2018, manteve-se no mesmo patamar entre 2018 e 2020 e voltou a aumentar em 2021. A queda abrupta em 2018 pode ter sido influenciada por vários fatores, como mudanças nas políticas de notificação, alterações nos critérios de registro ou variações nas práticas de atendimento. A implementação de políticas mais robustas, como o uso do Disque 100, pode ter contribuído para uma coleta mais sistemática dos dados de violência contra a população LGBTQIAPN+. É importante ressaltar que essa redução no número de denúncias não necessariamente reflete uma diminuição real na violência, podendo estar associada à subnotificação, na qual muitos casos permanecem não reportados devido ao medo de represálias ou à falta de confiança nas autoridades. 

Em 2020, as medidas de controle da covid-19, como o distanciamento social e o confinamento, podem ter afetado tanto a notificação quanto a incidência de violência. A pandemia trouxe aumento do estresse, isolamento social e vulnerabilidades econômicas, fatores frequentemente associados ao aumento da violência por parceiros íntimos.^21-23^ Embora as restrições de movimento possam ter dificultado o acesso a serviços de apoio e a notificação de casos, o aumento observado em 2021 pode estar ligado à flexibilização das medidas de controle da covid-19, que permitiu uma maior visibilidade e notificação dos casos.^21-23^

A região Sudeste do Brasil, densamente povoada e urbanizada, apresenta maior quantidade de casos de violência praticada por parceiros íntimos. Apesar de ser a região mais rica economicamente do país, ela enfrenta altos índices de notificação de violência praticada por parceiros íntimos. Esse contraste revela que a riqueza econômica não necessariamente reduz a violência. Revela também que as desigualdades socioeconômicas regionais podem intensificar a vulnerabilidade de grupos específicos, aumentando a exposição à violência. Essa maior frequência acontece entre pessoas de 20 a 29 anos, negras, com ensino fundamental, mulheres transexuais e heterossexuais.^8,24^ Tal frequência também ocorre entre pessoas de 20 a 29 anos, negras, com ensino fundamental, mulheres transexuais e heterossexuais. Os marcadores sociais da diferença associados à maior incidência refletem padrões sistêmicos de discriminação, enquanto normas culturais arraigadas contribuem para um ambiente propício à violência.^7,19^

A alta concentração de casos notificados em certas regiões pode indicar maior visibilidade e eficiência nos sistemas de notificação, o que reflete um aumento na conscientização sobre os direitos das pessoas trans e maior disposição para relatar casos de violência. Tem-se como exemplo a região Sudeste: com um número elevado de notificações, pode estar mais avançada em termos de reconhecimento e relato de casos de violência perpetrada por parceiros íntimos. Essa situação evidencia a necessidade de implementar estratégias específicas e adaptadas para enfrentar tal violência contra pessoas trans, especialmente em regiões com alta taxa de notificações.^25,26^

Quando os marcadores foram avaliados separadamente, observou-se uma diferença estatística na frequência dos episódios de repetição da violência praticada por parceiros íntimos contra pessoas transexuais. No Sul, destacou-se maior frequência de repetição dos casos, especialmente entre aqueles com ensino fundamental e em pessoas com deficiência física ou transtorno mental. Também foi notada uma disparidade nos grupos de pessoas do Norte, de origem amarela ou indígena e lésbicas que foram menos encaminhadas. 

A concentração desses casos nessas duas regiões brasileiras sugere peculiaridades geográficas, o que indica possíveis variações nas políticas de prevenção e redes de suporte.^8,24^ Isso revela as limitações nos recursos disponíveis nessa área ou para obstáculos geográficos que dificultam o acesso aos serviços de apoio. Destacam-se, também, desigualdades no acesso a serviços de apoio e na percepção da gravidade dos casos pelos profissionais envolvidos.^25-27^

O predomínio de maior repetição entre pessoas com ensino fundamental denota os desafios específicos enfrentados por indivíduos com níveis educacionais mais baixos, como menor conscientização sobre direitos, dificuldades em buscar ajuda e exposição prolongada a ambientes propícios à violência.^19^

A menor frequência de encaminhamentos para pessoas de origem amarela ou indígena pode refletir os preconceitos culturais, a falta de compreensão das necessidades específicas desses grupos ou a ausência de serviços culturalmente sensíveis.^19,27-29^ A frequência reduzida de encaminhamentos de pessoas lésbicas sugere possíveis lacunas na identificação e resposta às necessidades específicas dessa comunidade. Isso indica a necessidade de sensibilização sobre as formas específicas de violência que as afetam ou a presença de discriminação com base na orientação sexual.^30^

Esses padrões ressaltam a urgência de estratégias que promovam equidade no acesso aos serviços de apoio. Isso inclui a capacitação de profissionais, a implementação de serviços culturalmente sensíveis e a conscientização sobre as diferentes formas de violência enfrentadas por esses grupos específicos.^25-27^ Superar essas barreiras é essencial para garantir que todos recebam o suporte adequado diante de casos de violência por parceiros íntimos.

No estudo, foram examinados cinco perfis distintos de pessoas transgênero, cada um caracterizado por diferentes atributos sociodemográficos. Observou-se que todos os perfis apresentaram mais episódios de repetição da violência do que não repetição, destacando-se o perfil C, que inclui mulheres trans, sudestinas, negras, com ensino fundamental e lésbicas, como o que teve mais episódios de repetição.

A análise revela variações na relação entre as características individuais e a experiência de violência de repetição. O destaque para o perfil C sugere que a interseccionalidade das opressões desempenha um papel significativo.^7,18-20^ Isso indica que combinações específicas de identidades, como ser mulher trans, sudestina, negra, com ensino fundamental e lésbica, podem estar associadas à maior vulnerabilidade à repetição da violência. As experiências de discriminação e violência podem ser intensificadas quando múltiplos marcadores sociais da diferença se interseccionam. Isso evidencia a complexidade das dinâmicas sociais e a necessidade de abordagens mais sensíveis e inclusivas nas intervenções e políticas de combate à violência.^7,18-20^

Observou-se que todos os perfis foram encaminhados para outros pontos da rede de assistência à vítima de violência. O perfil A, que consiste em uma mulher trans, sudestina, de 20 a 29 anos, negra, com ensino médio, sem deficiência física ou transtorno mental e heterossexual, foi o que mais recebeu encaminhamentos.

Essa constatação indica que o perfil A teve maior propensão a ser encaminhado para serviços de apoio ou orientação. Isso pode ser atribuído a uma combinação específica de fatores, como a educação mais elevada, as características geográficas e a ausência de deficiência física ou transtorno mental, que podem influenciar a percepção dos profissionais sobre a necessidade de encaminhamento.^18,19^ A compreensão dessas especificidades é crucial para desenvolver estratégias mais eficazes de encaminhamento e suporte, garantindo que a assistência seja adequada e sensível às necessidades específicas de cada perfil de pessoa trans.

A confirmação de que não houve diferença significativa entre os perfis em relação à frequência da repetição da violência e dos encaminhamentos dos casos notificados é um resultado relevante. A análise de regressão logística binária não encontrou diferenças estatisticamente significativas na probabilidade de reincidência dos casos notificados. Esse resultado reforça a complexidade do fenômeno e indica que fatores associados à repetição da violência não variam de maneira significativa entre os diferentes perfis analisados.

Essa ausência de diferenças significativas sugere que as características individuais e contextuais consideradas nos perfis não são determinantes isolados para a repetição da violência. Isso indica a necessidade de uma abordagem holística ao compreender e lidar com a violência por parceiros íntimos contra pessoas trans.^3,29^

A complexidade do fenômeno indica que outros fatores, não abordados nos perfis, podem ter influência crucial na repetição da violência. Elementos como apoio social, acesso a recursos econômicos e discriminação sistêmica são mencionados como variáveis que desempenham papéis importantes.^21,29^ A interpretação dos resultados destaca a importância de considerar uma gama mais ampla de variáveis na compreensão e prevenção da violência repetida contra pessoas trans. A abordagem holística é fundamental para desenvolver estratégias mais eficazes, o que abrange características individuais e os diversos fatores contextuais que moldam a dinâmica da violência por parceiros íntimos.^3^

Este estudo, embora limitado pelos dados agregados e vieses nos registros administrativos, é um dos primeiros a abordar a violência por parceiros íntimos contra a população transgênero durante a pandemia de covid-19.^21^ As questões políticas e sociais das pessoas LGBTQIAPN+ continuam desafiadoras, com aumento e variação nos casos de violência por parceiros íntimos ao longo dos anos, influenciados por políticas de notificação e pela pandemia. Disparidades regionais destacam a necessidade de abordagens específicas, e a falta de diferenças significativas entre os perfis de repetição da violência e encaminhamentos aponta para a importância de uma abordagem holística que considere apoio social e discriminação sistêmica. O estudo contribui para a compreensão inicial do fenômeno e reforça a necessidade de pesquisas contínuas e intervenções sensíveis às suas complexidades.
